# Relationship Between Changes in Brain Natriuretic Peptide Levels After Initiation of Peritoneal Dialysis and Duration of Peritoneal Dialysis Monotherapy: A Retrospective Observational Study

**DOI:** 10.7759/cureus.86126

**Published:** 2025-06-16

**Authors:** Shoko Ohno, Hideki Yokoi, Kanna Shinkawa, Shingo Fukuma, Motoko Yanagita

**Affiliations:** 1 Department of Nephrology, Graduate School of Medicine, Kyoto University, Kyoto, JPN; 2 Department of Nephrology, Kumamoto University Graduate School of Medical Sciences, Kumamoto, JPN; 3 Department of Epidemiology, Disease Control and Prevention, Hiroshima University Graduate School of Biomedical and Health Sciences, Hiroshima, JPN; 4 Institute for the Advanced Study of Human Biology (ASHBi), Kyoto University, Kyoto, JPN

**Keywords:** brain natriuretic peptide (bnp), end-stage renal failure, fluid overload, maintanence hemodialysis, peritoneal dialysis (pd)

## Abstract

Background

Peritoneal dialysis (PD) is an integral modality in renal replacement therapy. However, inadequate fluid volume control is a key reason for PD withdrawal. Brain natriuretic peptide (BNP) is effective for evaluating fluid volume in PD patients. This study investigates whether BNP improvement after PD initiation reflects enhanced fluid control and correlates with longer PD duration.

Methods

A single-center retrospective observational study analyzed 99 patients who started PD between November 2002 and March 2022. BNP levels at PD initiation (BNP_0M_), and 6 months later (BNP_6M_) were used to calculate the BNP_6M_ /BNP_0M_ ratio. Patients were divided into BNP-improved and unimproved groups. PD duration was analyzed using the Kaplan-Meier method, and withdrawal risk from PD monotherapy was analyzed using a multivariable-adjusted Cox proportional hazards and nonlinear analysis.

Results

Seventy-one patients met the inclusion criteria, with a median follow-up period of 38 months. Kaplan-Meier analysis showed that the BNP-improved group had significantly longer PD monotherapy (log-rank test, P=0.0258). The Cox model indicated that a higher BNP_6M_/BNP_0M_ ratio increased withdrawal risk from PD monotherapy (P=0.038). In the Poisson regression models, improved BNP levels were associated with continued PD monotherapy.

Conclusion

Improvement in BNP levels after PD initiation correlates with extended PD monotherapy. Regular BNP monitoring as a fluid volume control marker may contribute to assessing stable PD periods and improving the quality of life of patients.

## Introduction

According to recent estimates, more than 272,000 individuals worldwide receive peritoneal dialysis (PD), representing 11% of the global dialysis population [1]. In Japan, it is estimated that over 13 million individuals are affected by various stages of chronic kidney disease (CKD), with more than 340,000 individuals dependent on dialysis modalities for the treatment of end-stage kidney disease (ESKD) [2, 3]. The prevalence of PD is approximately 3% in Japan. PD is an essential renal replacement therapy for ESKD and offers numerous advantages, including a reduced frequency of hospital visits, shorter periods of hospitalization, fewer dietary restrictions, diminished impact on hemodynamics, and better preservation of residual kidney function. Currently, in Japan, as a shared decision-making approach, it is becoming more prevalent, aiming to facilitate collaboration between patients and healthcare professionals in determining treatment and care plans that align with patients’ values and preferences while remaining professionally acceptable, and the indications of PD may expand. Furthermore, with the recent demographic shift towards an aging population, concepts such as last PD and assisted PD for elderly patients have emerged, further broadening the range of indications for PD. In this expanding trend of indications, it is imperative to manage patients in a manner that enables them to maintain a stable and sustainable PD to maintain their quality of life.

Fluid volume control is crucial for maintaining stability in peritoneal dialysis, as volume overload is associated with cardiac dysfunction [4, 5], inflammation [6], and mortality [7]. Mizuno et al. reported dialysis failure/ultrafiltration failure and peritonitis as primary causes of withdrawal from PD [8]. Kawaguchi et al. reported that ultrafiltration failure or inadequate adherence to salt and fluid restrictions was the most significant factor in 34% of 170 patients transitioning from PD to hemodialysis (HD) [9]. In the clinical management of PD patients, brain natriuretic peptide (BNP) is utilized as a measure to assess cardiac function and fluid volume. Clinical observations indicate that the initiation of peritoneal dialysis results in effective control of fluid volume, accompanied by stable continuation of PD in patients with improved BNP levels. The precise role of BNP in PD patients remains incompletely elucidated; however, studies suggest that BNP and N-terminal pro BNP (NT-proBNP) are valuable for the diagnosis of systolic heart failure in PD patients [10] and that BNP may function as a potential indicator of fluid volume in this patient population [11]. Nevertheless, limited research has been conducted to investigate the association between BNP levels and the prognosis of patients undergoing PD.

In the present study, we investigated whether an improvement in BNP levels following PD initiation is associated with a longer duration of subsequent PD monotherapy.

## Materials and methods

Study design and setting

We conducted a single-center retrospective observational study. The study population comprised patients who initiated PD at 18 years of age or older at the Kyoto University Hospital between November 2002 and March 2022. The hospital is a 1141-bed tertiary general hospital with 34 divisions, including internal medicine, surgery, emergency department, and psychiatry, which manages both acute and chronic conditions of various diseases.

**Figure 1:**
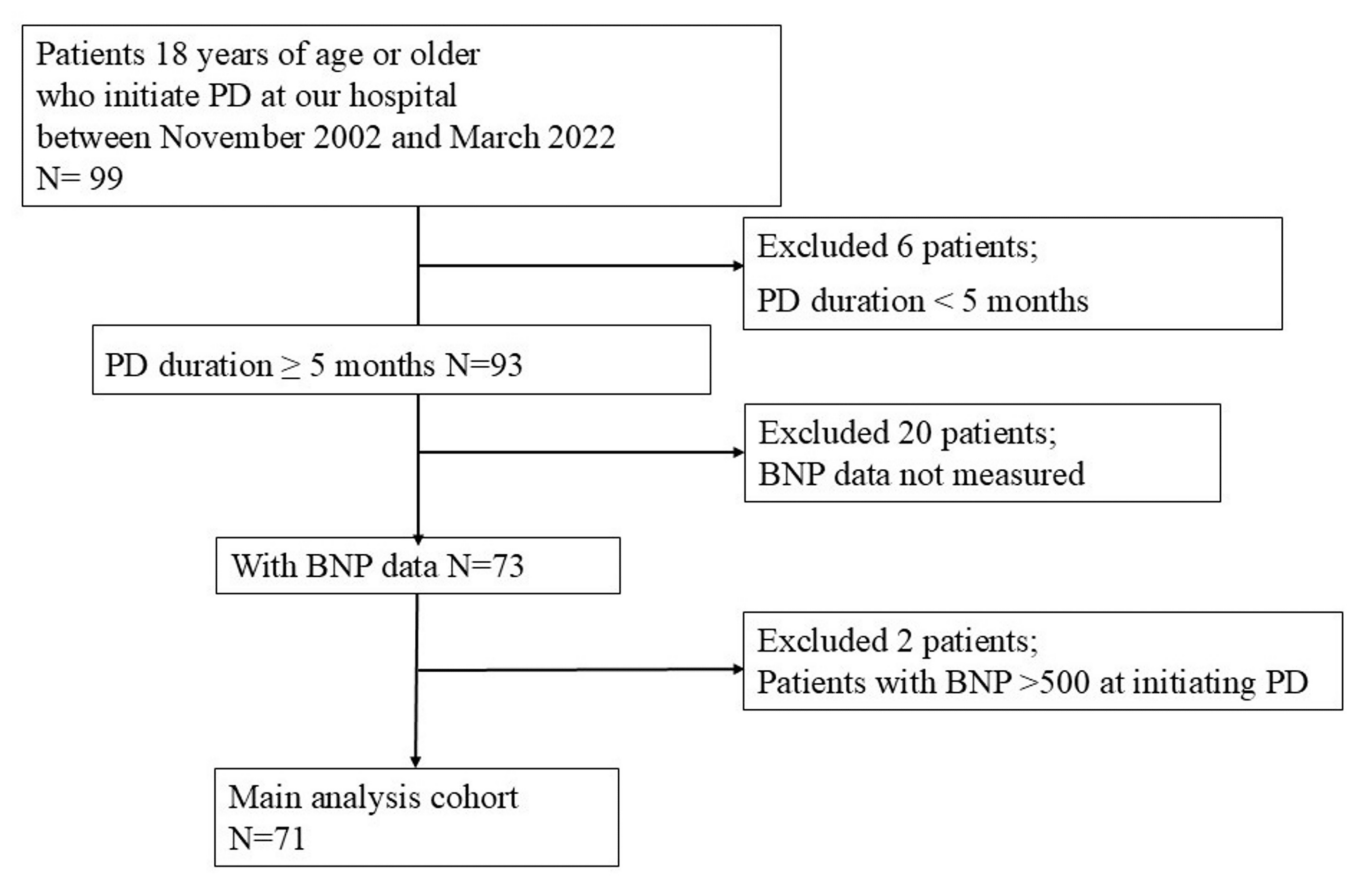
Flow of Patients in the Study Cohort. Abbreviations: PD, peritoneal dialysis; BNP, brain natriuretic peptide.

 The exclusion criteria, defined a priori, are delineated in the patient flow diagram (Figure 1). 

Six patients were excluded because their PD duration was less than 5 months. Twenty patients were excluded because their BNP data were not measured at PD initiation and/or 6 months after PD initiation. Two patients were excluded because their BNP values exceeded 500 at PD initiation, as these were considered outliers. The final study sample comprised 71 participants. Mortality, transition to HD, and transition to hybrid therapy with HD were considered as events. These events were also collectively defined as a composite outcome and analyzed as a composite event. This study was approved by the Ethics Committee of the Kyoto University Graduate School and Faculty of Medicine (R1718). The Ethical Guidelines for Medical and Health Research Involving Human Subjects in Japan, on which committee approval is based, do not require informed consent from patients because of the secondary use of existing patient data collected for clinical purposes. Patient recruitment was conducted using an appropriately managed opt-out method, as mandated by the aforementioned guidelines, and documents outlining the patients' rights to decline participation were disclosed. Personal information was managed using secure computers in accordance with the guidelines of the Personal Information Protection Law in Japan. 

Data collection

The medical records were de-identified. Clinical data, such as age, sex, complications, cause of ESKD, blood pressure (BP), blood tests, and urinalysis results, were collected from the medical records. Heart failure, arrhythmia, and ischemic heart disease insurance disease registry collected from medical records. Heart failure, arrhythmia, and ischemic heart disease insurance disease registry collected from medical records. Ejection Fraction (EF) data were collected from echocardiograms performed within a year before and after PD initiation in 43 of 71 cases. "Cardiothoracic ratio (CTR) after PD initiation" in Table 1 was measured from chest radiograph within 1 month before and 6 months after PD initiation (6 months; n =32). Peritoneal equilibration test (PET) data were collected in 53 of 71 cases. PD initiation was defined as the initial PD fluid retention. BNP levels were measured using an automated electrochemiluminescence immunoassay at our hospital. Data collection from PD patients was performed during the introduction period and 6 months after PD initiation. Of the BNP levels measured within one month before and after the initiation of PD, the BNP levels closest to the initiation date were defined as BNP_0M_. Similarly, BNP levels were measured within one month before and after 6 months after the initiation of PD, and the BNP level closest to 6 months was defined as BNP_6M_. The patients were grouped according to the ratio of BNP_6M_ /BNP_0M_. Patients with the ratio of BNP_6M_ /BNP_0M_ < 1 were defined as the “BNP improved group” and ≥1 as “BNP unimproved group.” In Kaplan-Meier analysis, comparisons were also made among three groups: BNP_6M_ /BNP_0M_ <0.8, 0.8≤ BNP_6M_ /BNP_0M_ <1.2, and BNP_6M_ /BNP_0M_ ≥1.2. The end of follow-up was defined as 120 months.

Statistical analysis

The outcomes were mortality, transition to HD, and transition to hybrid therapy with HD, which were analyzed using time-to-event analyses. Outcome observation was started after the BNP_6M_ measurement. Kaplan-Meier survival curves were used for PD duration, and log-rank statistics were used to assess the differences between the groups classified by the BNP_6M_ /BNP_0M_ ratio. The risk of withdrawal from PD monotherapy was analyzed using a multivariable-adjusted Cox proportional hazards model; the hazard ratio and 95% confidence interval (CI) were analyzed with withdrawal from PD monotherapy as the outcome. Age, sex, nephrosclerosis, and diabetic nephropathy were used as covariates. Model 1 was adjusted for age and sex; model 2 was additionally adjusted for nephrosclerosis; and model 3 was further adjusted for diabetic nephropathy. Finally, the relationship between the ratio of BNP_6M_ /BNP_0M_ and the incidence of withdrawal from PD monotherapy was analyzed using Poisson regression. The findings are presented as incidence rates with 95% confidence intervals. Continuous variables were presented as mean (standard deviation (SD)) or median (interquartile range (IQR)). Values of p < 0.05 were considered significant. Statistical analyses were performed using EZR version 1.64 (Jichi Medical University, Tochigi, Japan) and R version 4.1.2 (R Foundation for Statistical Computing, Vienna, Austria).

## Results

From November 2002 to March 2022, we identified 99 patients who initiated PD at our hospital. After excluding 28 patients whose PD duration was less than 5 months, whose BNP data were not measured, or whose BNP was >500 pg/mL at PD initiation, 71 patients were included in the main analysis cohort (Figure 1).

The characteristics of patients in the “BNP improved group” and the “BNP unimproved group” are presented in Table 1.

**Table 1:** Characteristics of the patients Values represent medians with interquartile ranges (IQR: 25%, 75%). Of the BNP levels measured within one month before and after the initiation of PD, the BNP levels closest to the initiation date was defined as BNP_0M_. Similarly, BNP levels were measured within one month before and after 6 months after the initiation of PD, and the BNP level closest to 6 months was defined as BNP_6M_. Abbreviations: BMI, body mass index; BNP, brain natriuretic peptide; BP, blood pressure; CTR, cardiothoracic ratio; CRP, C-reactive protein; EF, ejection fraction; PET, peritoneal equilibration test; PD, peritoneal dialysis, BNP_0M_, BNP levels at peritoneal dialysis initiation; BNP_6M_, BNP levels 6 months after initiation. ^$^BNP improved (n = 38), BNP unimproved (n = 15), ^†^BNP improved (n = 22), BNP unimproved (n = 10), ^#^BNP improved (n = 25), BNP unimproved (n = 18).

Characteristics	BNP improved (BNP_6M _/BNP_0M _< 1)	BNP unimproved (BNP_6M _/BNP_0M _> 1)	P-value
N	44	27	
Diabetes, n(%)	5 (11.4)	9 (33.3)	0.033
Nephrosclerosis, n (%)	13 (29.5)	2 (7.4)	0.036
Heart failure, n (%)	10 (22.7)	4 (14.8)	0.544
Arrhythmia, n (%)	3 (6.8)	0 (0)	0.283
Ischemic heart disease, n (%)	3 (6.8)	1 (3.7)	1
Male sex, n (%)	21 (47.7)	15 (55.6)	0.627
Age, yr	61.0 (47.25, 66.0)	55.0 (44.0, 62.5)	0.292
BMI, kg/m^2^	21.8 (19.4, 23.8)	21.8 (19.5, 26.2)	0.522
BMI after PD initiation, kg/m^2^	22.0 (20.4, 24.1)	22.0 (20.1, 25.5)	0.631
CTR (%)	46.8 (44.0, 51.3)	47.4 (41.9, 51.1)	0.808
CTR after PD initiation, %^†^	45.1 (44.0, 48.9)	49.5 (46.4, 51.1)	0.258
EF, %^#^	68.8 (65.9, 80.3)	66.9 (64.1, 80)	0.858
Hemoglobin, g/dl	9.4 (8.8, 10.3)	9.5 (9.0, 10.4)	0.59
Albumin, mg/dl	3.5 (3.3, 3.8)	3.5 (3.0, 3.8)	0.622
Creatinine, mg/dl	8.8 (7.1, 9.9)	9.2 (7.6, 10.6)	0.3
Urea nitrogen, mg/dl	74.0 (63.0, 87.8)	78.5 (66.3, 106.5)	0.238
CRP, mg/dl	0.15 (0.0, 0.58)	0.25 (0.1, 0.95)	0.317
Total cholesterol, mg/dl	175.0 (139.0, 200.3)	162.5 (131.8, 203.0)	0.675
Systolic BP, mmHg	141.5 (124.8, 152.3)	139.0 (126.0, 155.0)	0.794
Diastolic BP, mmHg	79.0 (74.8, 87.3)	86.0 (73.0, 93.0)	0.41
Peritonitis, n	0.0 (0.0, 1.0)	0.0 (0.0, 0.0)	0.205
Category High in PET, n (%)^$^	10 (26.6)	5 (33.3)	0.48
Composite event, n (%)	25 (56.8)	20 (74.1)	0.205
HD, n (%)	12 (48)	7 (35)	
Hybrid therapy, n (%)	12 (48)	9 (45)	
Death, n (%)	1 (4)	4 (20)	

Among the participants, the median age was 61 years (IQR 47.25-66.0) in the "BNP improved group" and 55 years (IQR 44.0-62.5) in the "BNP unimproved group.” No statistically significant differences were observed between the two groups. The prevalence of heart failure, arrhythmia, and ischemic heart disease showed no significant variation between the "BNP improved group" and the "BNP unimproved group." Similarly, the ejection fraction (EF) did not exhibit a significant difference between these groups. There was no significant difference in CTR and BMI between the two groups at the time of PD initiation or six months after PD initiation. Sex was similar between the two groups. Hemoglobin, albumin, creatinine, urea nitrogen, and CRP levels were not significantly different between the two groups. The percentage of patients who showed the "High" category in the first PET after PD initiation was not increased in the BNP unimproved group. Diabetes as a comorbidity exhibited a significantly higher prevalence in the BNP unimproved group (nine of 27 patients, 33.3% (P=0.033)), whereas nephrosclerosis was significantly more prevalent in the BNP improved group (13 of 44 patients, 29.5% (P=0.036)). The causes of end-stage renal disease in 71 patients who initiated PD are presented in Table 2.

**Table 2:** Causes of end-stage renal disease in the 71 patients who initiated PD Abbreviations: PD, peritoneal dialysis; ANCA, Anti-neutrophil cytoplasmic antibody.

Disease	n	%
Chronic glomerulonephritis (Other than those diagnosed as IgA by renal biopsy)	17	23.94
IgA nephritis	14	19.72
Nephrosclerosis	13	18.31
Diabetic nephropathy	13	18.31
ANCA associated vasculitis	2	2.82
Polycystic kidney	1	1.41
Lupus nephritis	1	1.41
Interstitial nephritis	1	1.41
Other	9	12.68

Fourteen patients were diagnosed with IgA nephropathy via renal biopsy, and 17 patients were diagnosed with chronic glomerulonephritis other than IgA nephropathy, collectively accounting for 31 patients (43%). Compared to the common causes of dialysis initiation, the rates of diabetic nephropathy and nephrosclerosis tended to be lower. Among the 71 patients who fulfilled the inclusion criteria, 45 (63.4%) withdrew from PD monotherapy during a median follow-up of 38 months. The causes of withdrawal from PD monotherapy in the present study are presented in Table 3.

**Table 3:** Causes of withdrawal from PD monotherapy Abbreviations: EPS, encapsulating peritoneal sclerosis; PD, peritoneal dialysis.

Cause	n	%
Ultrafiltration failure	16	35.6
Insufficient solute removal	11	24.4
Peritonitis	9	20.0
Death	5	11.1
Prevention of EPS	2	4.4
Other	2	4.4

Ultrafiltration failure was the predominant cause of withdrawal from PD monotherapy in 16 patients (35.6%). The second most frequent cause was insufficient solute removal, affecting 11 patients (24.4 %), while peritonitis constituted the third most common cause, affecting nine patients (20.0%).

**Figure 2:**
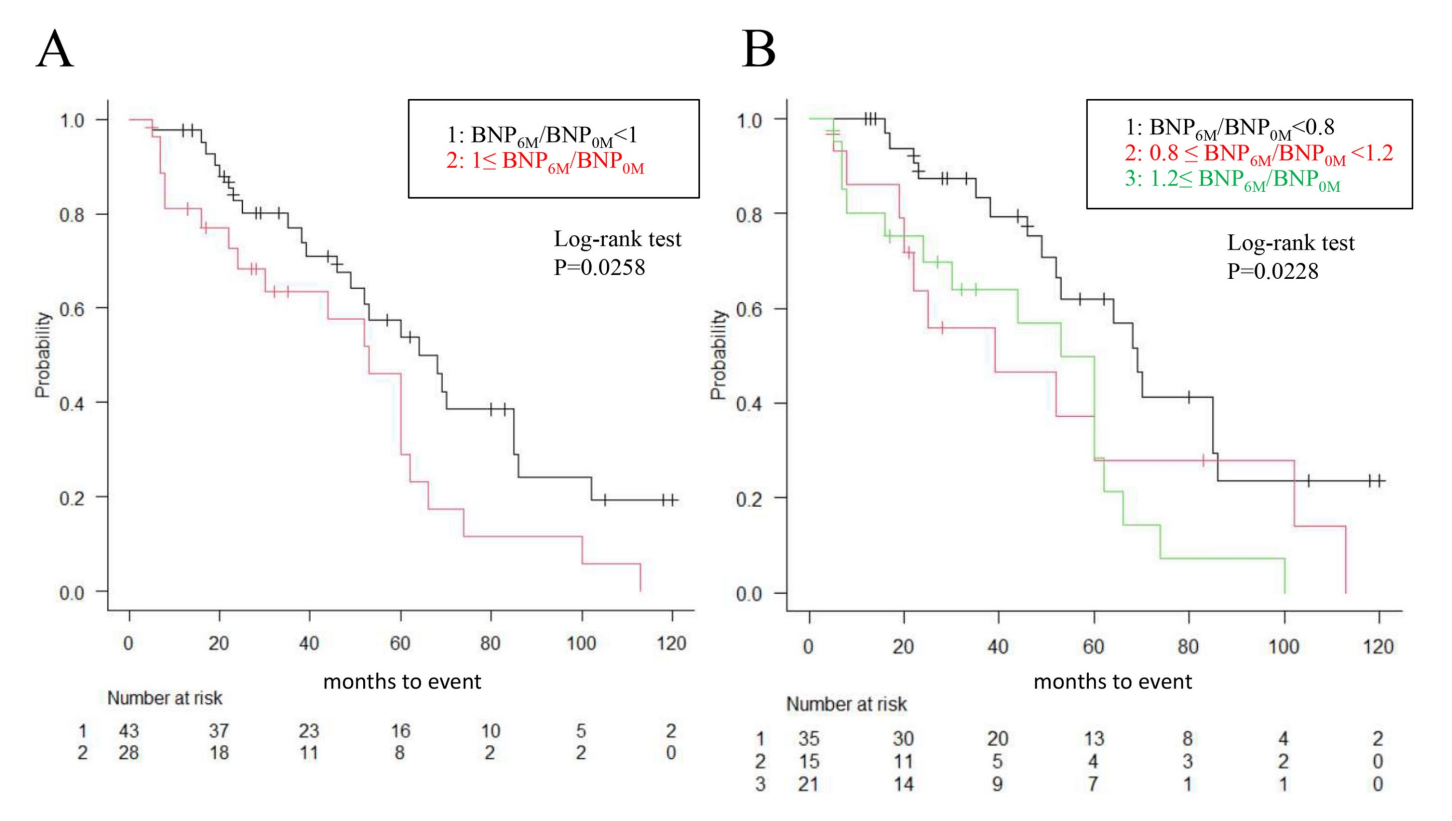
Kaplan-Meier analysis of PD monotherapy duration. Kaplan-Meier survival curves were used for PD monotherapy duration, and log-rank statistics were used to evaluate the differences between groups classified by BNP_6M_ /BNP_0M_ ratio. PD monotherapy duration was significantly prolonged in the BNP improved group than in the BNP unimproved group, both in the two-group analysis and in the three-group analysis. Abbreviations: PD, peritoneal dialysis; BNP, brain natriuretic peptide, BNP_0M_, BNP levels at peritoneal dialysis initiation; BNP_6M_, BNP levels 6 months after initiation.

PD monotherapy duration was analyzed using the Kaplan-Meier method (Figure 2).

Initially, patients were stratified into two groups based on whether the BNP_6M_/BNP_0M_ ratio was less than 1 or greater than 1. The PD monotherapy duration was significantly longer in the BNP improved group; the ratio of BNP_6M_/BNP_0M_ was less than 1 (Figure 2A; log-rank test P=0.0258). Subsequently, patients were categorized into three groups according to the BNP_6M_/BNP_0M_ ratio (BNP_6M_/BNP_0M_ < 0.8, 0.8≤ BNP_6M_/BNP_0M_ < 1.2, and BNP_6M_/BNP_0M_ ≥1.2). The group exhibiting the most substantial improvement in BNP (the ratio of BNP_6M_/BNP_0M_ was less than 0.8) exhibited the longest duration of PD monotherapy (Figure 2B; log-rank test P=0.0228).

Furthermore, in the multivariable-adjusted Cox proportional hazards model adjusted for age and sex, the ratio BNP_6M_/BNP_0M_ was associated with a significantly increased risk of withdrawal from PD monotherapy (P=0.038) (Table 4).

**Table 4:** Cox multivariate analysis The risk of withdrawal from PD monotherapy was analyzed using a multivariable-adjusted Cox proportional hazards model. The hazard ratio (HR) and 95% confidence interval (CI) were analyzed using withdrawal from PD monotherapy as the outcome. Abbreviations: BNP, brain natriuretic peptide.

Factor	Hazard ratio	P-value
BNP unimproved	2.25 (1.17-4.34)	0.038
Age	1.01 (0.99-1.04)	0.548
Sex	0.90 (0.48-1.70)	0.471

The ratio BNP_6M_/BNP_0M_ maintained its association with incident withdrawal from PD monotherapy after multivariable adjustment (Table 5).

**Table 5:** Cox multivariate analysis The risk of withdrawal from PD monotherapy was analyzed using a multivariable-adjusted Cox proportional hazards model. The hazard ratio (HR) and 95% confidence interval (CI) were analyzed using withdrawal from PD monotherapy as the outcome. Age, gender, nephrosclerosis and diabetic nephropathy were set as covariates. Model 1 adjusted for age and sex; model 2 additionally adjusted for nephrosclerosis; and model 3 further adjusted for diabetic nephropathy.

Model	Adjustment for covariates	Hazard ratio (95% CI)	P-value
1	Age and Sex	1.90 (1.04-3.49)	0.0375
2	+Nephrosclerosis	1.82 (0.96-3.43)	0.0656
3	+Diabetic Nephropathy	1.71 (0.91-3.20)	0.0968

The ratio BNP_6M_ /BNP_0M_ attenuated the association with incident withdrawal from PD monotherapy after multivariable adjustment for nephrosclerosis and diabetic nephropathy (Table 5).

**Figure 3:**
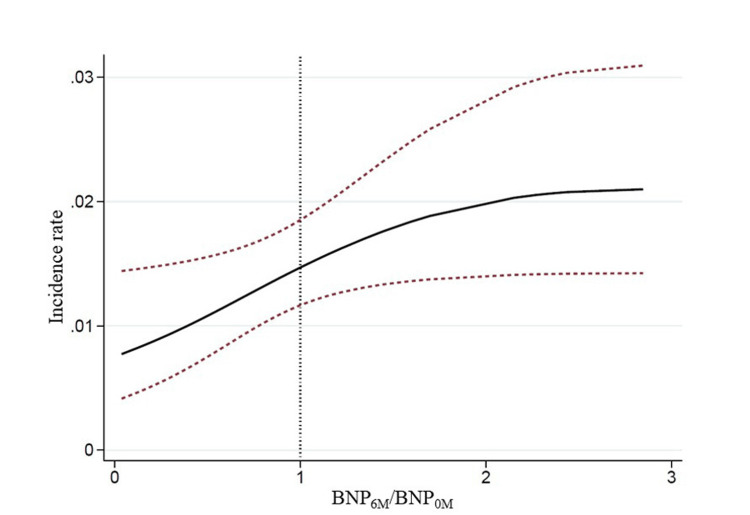
Poisson regression models. The relationship between the ratio of BNP_6M_/BNP_0M_ and the incidence of withdrawal from PD monotherapy was analyzed using Poisson regression. The findings were presented as incidence rate at 95% confidence intervals. The incidence of withdrawal from PD monotherapy increased as the BNP6M/BNP0M ratio increased. This relationship exhibited a nearly linear trend up to a BNP_6M_/BNP_0M_ ratio of approximately 2. Abbreviations: PD, peritoneal dialysis; BNP, brain natriuretic peptide; BNP_0M_, BNP levels at peritoneal dialysis initiation; BNP_6M_, BNP levels 6 months after initiation.

Finally, in Poisson regression models, we evaluated the association between the BNP_6M/_BNP_0M_ ratio and the incidence of withdrawal from PD monotherapy (Figure 3).

Figure 3 shows that the incidence of withdrawal from PD monotherapy increased as the BNP_6M_/BNP_0M _ratio increased. This relationship exhibited a near-linear trend up to a BNP_6M_/BNP_0M_ ratio of approximately 2.

## Discussion

In the present study, we investigated the association between improvement in BNP levels following PD initiation and prolonged PD monotherapy duration. The Kaplan-Meier analysis revealed that the PD monotherapy duration was significantly longer in the BNP improved group than in the BNP unimproved group, both in the two-group and in the three-group analyses. These findings suggest that a longer PD monotherapy duration is achieved when BNP levels improve with the initiation of PD. The multivariate analysis revealed a relationship between BNP improvement and longer PD monotherapy duration after demonstrating a correlation between BNP improvement and extended PD monotherapy duration after adjusting for age and sex. In the Poisson regression models, a significant trend was observed between the BNP_6M_/BNP_0M_ ratio and the incidence of withdrawal from PD monotherapy; as BNP levels improved, PD continuation became more probable. This result, in conjunction with the Kaplan-Meier analysis, indicates an association between improvement in BNP levels following PD initiation and prolonged PD monotherapy duration.

Causes of PD withdrawal include peritonitis, insufficient solute removal, and poor volume control. Inadequate volume control is one of the most significant factors [8, 9]. Although body weight, edema, blood pressure (BP), and cardiothoracic ratio (CTR) have been used as indicators of fluid volume, this study focused on BNP, which is secreted by cardiomyocytes in response to ventricular hypertrophy, inflammation, or distention of the left ventricle, and myocardial injury [12]. The production and secretion of BNP are increased in heart failure, and plasma BNP levels are significantly elevated in congestive heart failure [13]. The significance of plasma BNP levels has been established for the diagnosis of heart failure, assessment of severity, and prognostic prediction [14]. BNP is synthesized as a prehormone consisting of 108 amino acids. It is cleaved in equal proportions into the biologically active 32 amino acid BNP, which represents the C-terminal fragment, and the biologically inactive 76 amino acid N-terminal fragment (NT‐proBNP) [15]. BNP is eliminated by binding to the clearance receptor (natriuretic peptide receptor) or by neutral endopeptidase (NEP) [16]. Conversely, NT-proBNP is primarily eliminated via glomerular filtration. The relationship between metabolism and pathology remains incompletely elucidated; however, the clearance of both is affected by reduced renal function, NT-proBNP being more significantly impacted [17]. In the present study, BNP was used rather than NT-proBNP because BNP represents the active form and more accurately reflects the physiological action of BNP.

Regarding the elevated plasma concentration of BNP as renal function deteriorates, it has been suggested that a chronic volume overload may be implicated. In patients undergoing dialysis, BNP levels have been observed to be higher pre-dialysis and lower post-dialysis [18, 19]. The reduction in BNP plasma concentrations following HD may be attributable to the decreased production/secretion of BNP resulting from a reduction in plasma volume, elimination by dialysis, or a combination of these factors. The molecular weight of BNP is 3.5 kDa, rendering it theoretically removable by dialysis [20]. BNP has been demonstrated to be a prognostic predictor of all-cause mortality, including cardiovascular mortality, in the studies of patients with CKD, particularly those undergoing HD [19, 21]. While no standard cut-off value for BNP levels in HD patients has been established, evidence suggests that it can serve as a valuable tool for dry weight assessment; furthermore, there are reports that suggest that BNP levels in HD patients have limitations in their utility for fluid volume assessment [22, 23]. The literature on PD patients is notably scarce. Research has demonstrated that BNP levels in PD patients are markedly lower than those observed in HD patients, suggesting a diminished cardiac burden. [24].

Evidence suggests that BNP levels may serve as an indication of fluid volume in PD patients. Joo Hoon Lee et al. examined the changes (Δ) of body weight (BWt), BP, and serum BNP at the initiation of PD and during subsequent follow-up periods in 30 hypertensive children undergoing PD. The results showed that Δ diastolic blood pressure (DBP) and ΔBNP were significantly higher in children with hypertension than in the controls. The findings indicated that ΔDBP and ΔBNP dropped significantly after reduction of ΔBWt and that ΔBNP exhibited a significant correlation with Δ systolic blood pressure (SBP) and ΔDBP [11]. The methodology for determining the dry weight is well established for HD, and PD adheres to these principles. Indeed, in Japanese peritoneal dialysis guidelines, dry weight is currently determined clinically with reference to the absence edema, the absence pleural effusion or pulmonary congestion on chest X-Ray, CTR <50%, atrial natriuretic peptides (ANP) 50-100 pg/mL, inferior vena cava diameter and respiratory variability [25]. In recent years, bioimpedance spectroscopy has gained attention as an alternative approach to assess fluid status. However, Tan et al reported that in a prospective, randomized, open-label, blinded trial of 308 PD patients, the use of bioimpedance devices did not significantly improve the guidance of fluid management and PD prescription compared to clinical assessment alone [26]. Fluid volume assessment in PD patients is sometimes difficult. However, the present study showed that BNP_6M_/BNP_0M_ could be a new and essential indicator when assessing fluid volume in PD patients, along with other existing indicators such as BMI and CTR.

BNP levels exhibit considerable variability among individuals. Furthermore, BNP levels tend to increase in elderly populations, potentially due to age-dependent decreases in left ventricular compliance and renal function [27]. In the present study, the BNP ratio before and after the initiation of PD was utilized to make it relative, thereby mitigating the effects of individual differences and age.

In the present study, the observed correlation between BNP improvement and PD duration may be attributed to the fact that BNP levels reflect enhanced volume control and reduced cardiac load following PD initiation, suggesting that the PD monotherapy duration was extended in patients exhibiting superior volume control. Other volume control indices, such as CTR and BP, showed no significant difference in this study when compared between the BNP improved group and the BNP unimproved group. These findings indicate that BNP measurement serves as a valuable indicator of fluid volume control in PD patients and may contribute to the maintenance of stable PD treatment. Regarding patient characteristics, the higher prevalence of diabetic nephropathy in the BNP unimproved group suggests that patients with a diabetic nephropathy background are more likely to exhibit poor fluid volume control.

Several limitations are present in this study. First, this investigation is a single-center study with a relatively small sample size. Second, the extent to which BNP accurately reflects fluid volume in PD patients remains a subject of debate. CTR data after PD initiation were collected in only 32 of the 71 cases. Third, this evaluation of peritoneal function and residual kidney function was insufficient. No significant difference was observed in the proportion of patients categorized as high transporters on PET. However, only 54 of 71 patients underwent PET, and there was variability in the timing of PET administration. Further detailed analysis is needed to clarify the relationship between PET and BNP improvement.

## Conclusions

The findings of the present study indicate that an improvement in BNP levels following PD initiation is associated with an extended PD monotherapy duration. These results suggest that regular assessment of BNP as an indicator of fluid volume control may contribute to the maintenance of stable PD periods, which in turn may enhance the quality of life of patients. Further investigation is warranted to elucidate the relationship between BNP levels and the continuation of stable PD duration.
